# Observations on the Use of Arsenic in the Intermittent Fevers of a Tropical Climate; to Which Is Prefixed an Account of the Weather, at Sierra Leone, during the Season in Which Such Fevers Are Most Prevalent

**Published:** 1795

**Authors:** Thomas Masterman Winterbottom

**Affiliations:** Physician to the Settlement at Sierra Leone.


### MEDICAL FACTS

AND

### OBSERVATIONS.

I. *Observations on the Use of Arsenic in the Intermittent Fevers of a tropical Climate; to which is prefixed an Account of the Weather, at Sierra Leone, during the Season in which such Fevers are most prevalent. By* Thomas Masterman Winterbottom, *M. D. Physician to the Settlement at Sierra Leone.*

AS arsenic, though of late years frequently and successfully used in England for the cure of intermittent fevers, has not, to my knowledge, been hitherto employed in a tropical climate; some account of its use in Africa, with the histories of a few of the cases in which it was exhibited, will not, I hope, be altogether unacceptable.

It may be proper however to premife a fhort account of the weather at Sierra Leone during the feafon in which intermittents are moft prevalent.

The year may be divided into the rainy, tornado, and dry feafons. The rains on this part of the coaft commonly fet in about the end of May, or beginning of June; and continue, more or lefs violently, until the beginning or middle of September: they are then fucceeded by tornadoes, which continue until the end of November. It muft be obferved, however, that the rains are not only carried off by tornadoes, but alfo brought on by them; and that the tornadoes preceding the rains are, in general, lefs regular than thofe which terminate them. The dry feafon continues from December until May, though fhowers of rain fometimes occur during the dry months.

In 1792, the rains commenced about the end of May, and continued for fome time to be very heavy; from the middle of July, however, until the laft week of Auguft, there were frequent intervals of fair weather, twelve hours of rain being generally followed by twenty-four or thirty hours of fair weather, with fometimes a bright fun. During this period the thermometer at noon ufually ftood at from 78° to 80°.

The

The laſt week of Auguſt and firſt week of September were remarkable for an almoſt inceſſant rain, which was for the moſt part ſmall and drizzly, though it ſometimes fell in heavy ſhowers; the air at the ſame time felt cold and raw, particularly in the evenings and mornings, when a thick fog covered the hills. The thermometer at noon was from 77° to 80°.

On the 7th of September a tornado came on, which returned on the 10th, 15th, 16th, 18th, 19th, 21ſt, 22d, 24th, 26th, 28th, and 30th.

On the 8th, 11th, 12th, 25th, and 29th, the ſhowers of rain were frequent.

On the 9th, 11th, 14th, and 23d, thunder and lightning occurred during ſome part of the day. The 9th, 13th, 15th, 17th, 24th, and 26th were ſultry and almoſt calm. During the continuance of the rains, the winds chiefly blew from between the ſouth and weſt points, but moſt frequently from the ſouth-weſt, whence alſo the heavieſt rain came.

As ſoon as the tornadoes appeared, the ſea and land breezes had a more regular ſucceſſion; the ſea breeze uſually began from the northweſt about eight or nine A. M., and towards ſunſet drew round to the weſt: the land breeze then ſetting in from the eaſt or ſouth-eaſt, con-

 tinued tinued to blow all night and during the early part of the morning.

Towards the end of the month the thermometer generally ftood at 82° at noon, the atmofphere being lefs hazy, and the air cool.

The month of October was throughout attended with regular fea and land breezes; the atmofphere was free from haze, but fometimes overcaft with clouds during the day; the whole of the month was cool and agreeable, though the thermometer at noon generally ftood at 82°, and on the 29th at 84°.

A tornado occurred every night, or early in the morning, from the 1ft to the 18th inclufively, frequently attended with heavy rain for fome hours, and with much thunder and lightning. During the remainder of the month the tornadoes became lefs frequent, occurring only on the 19th, 21ft, 23d, 25th, 27th, 28th, and 29th. The 1ft, 17th, and 24th were fultry. On the 24th it was calm all day. On the 3d there was much thunder and lightning. On the 7th, 15th, 18th, 21ft, and 30th, frequent fhowers of rain fell. The tornado on the 17th came from the fouth-weft which is uncommon. The tornado on the 2d was not followed by rain. The 26th was remarkably hazy all day.

The

The lightning was extremely vivid on the 28th, appearing in long ſtreams or chains of fire.

The month of November was much warmer than the preceding one, the thermometer at noon being from 82° to 84°. On the 11th it roſe to 85°. It was on the 5th at 75°. There was continued rain till noon, when the ſky became clear, the day calm and ſultry. The atmoſphere during the greateſt part of the month was clouded and hazy, at leaſt the tops of the hills were covered with haze during ſome part of the day. The ſea and land breezes continued to blow very freſh, but the mornings were frequently calm and ſultry till near ten A. M. On the 28th it was calm all day. Tornadoes occurred on the 2d, 8th, 10th, 12th, 13th, 16th, 19th, and 25th. The 5th, 17th, and 23d were rainy. The 5th, 11th, 14th, 18th, and 28th, were ſultry, with a little wind.

In December alſo the ſky was generally hazy and clouded; the ſea and land breezes were pretty freſh during their continuance, but the mornings were for the moſt part calm, the ſea breeze not ſetting in till near ten A. M.; the evenings alſo were cloſe and ſultry from ſun-ſet till late at night.

A tornado came on, the morning of the 7th, followed by much rain, thunder, and lightning; but it cleared up before noon; a heavy ſhower fell in the afternoon of the ſame day.

The cleareſt days this month were the 3d, 9th, 13th, 18th, 24th, and 25th.

On the 5th, 8th, 14th, 15th, and 22d, gentle ſhowers fell: on the 8th there was much thunder and lightning. The weather was ſultry, with little wind, on the 1ſt, 3d, 14th, 19th, 22d, and 27th. The 14th and 27th were calm days. The land wind blew all day on the 13th, and the ſouth-weſt and ſouth-ſouth-weſt winds on the 2d, 30th, and 31ſt days. The thermometer at eight A. M. uſually ſtood at from 77° to 80°; on the 13th at 75°, and on the 26th at 81°: at noon it was from 81° to 84°; at eight P. M. from 78° to 80°.

The remittent fever which during the months of June, July, and Auguſt, had very generally prevailed here, and had raged with great violence, began to abate in the month of September. Early in the month, this diſeaſe had not only become leſs frequent, but alſo more mild in its ſymptoms, gradually changing into the form of an intermittent. Towards the end of the month it became very rare, the caſes which occurred being chiefly among the whites, eſ-

pecially pecially thoſe lately arrived in the country; or others who had been irregular and intemperate during the courſe of preceding intermittent complaints.

In the months of October, November, and December, intermittents were ſo prevalent, that ſcarcely a family in the ſettlement, although the whole number was nearly 400, remained perfectly free from them. They generally obſerved the quotidian and tertian type; there were, however, a few inſtances of double tertians. Moſt of the above caſes were ſo mild, particularly among the men, as not to prevent them from following their different occupations, except during the time of the paroxyſm. But in ſome inſtances, the daily recurrence of the diſeaſe, the long continuance of the paroxyſm, and a poor diet, conſiſting chiefly of ſalted meats, rice, caſſada, &c. reduced the patients to a ſtate of great debility, and inſenſibly laid the foundation of long and tedious complaints. The greateſt ſufferers from intermittents were thoſe who had previouſly laboured under remittent fevers, and had not yet recovered their ſtrength; alſo perſons of delicate and irritable habits, children, and women giving ſuck.

In every inſtance where the bark was taken in due quantities, and perſiſted in for a proper length of time, the paroxyſm was ſpeedily checked, and the danger of a relapſe effectually prevented; nor did the patient ſuffer thoſe ill effects which uſually occur where the diſeaſe has continued long, and been left to itſelf. Few, however, of the common people could be prevailed upon to take the bark in any form; and even thoſe who took enough of it to obviate the return of a ſingle paroxyſm, would ſeldom continue it a ſufficient length of time to eradicate the diſeaſe. Theſe conſiderations, joined to an apprehenſion that ſerious and alarming conſequences might enſue from frequent relapſes, determined me to try the effects of the mineral ſolution, according to the plan recommended by Dr. Fowler *. The fear of diſordering the bowels, and inducing dyſenteric ſymptoms, rendered me at firſt very cautious in its uſe; but on finding, after repeated trials, that no ill effects were produced by its exhibition, I was encouraged to employ it more generally. The ſucceſs with which it was attended will appear from the following detail of caſes:

* Medical Reports of the Effects of Arſenic in the Cure of Agues, &c. 8vo. London, 1786.

CASE

### CASE I.

October 4.—S. Peters, a black, aged four years, is affected every day, about noon, with coldneſs and violent ſhiverings, which continue near an hour, and are then ſucceeded by a hot dry ſkin, head-ach, and ſometimes vomiting. The paroxyſm is terminated in the evening by a copious perſpiration. In the abſence of the fit he makes no complaint, but appears languid and weak; and has little appetite. A conſiderable degree of hardneſs is felt on the left ſide, with a tumour projecting below the cartilages of the falſe ribs. He was ordered to take four drops of the mineral ſolution three times a day.

5. Had no cold fit yeſterday at the uſual time, but appeared heavy and uneaſy; no ſickneſs or griping was occaſioned by the drops.

8. Has had no return of the paroxyſm ſince the 3d. No griping nor any ſenſible effect has been produced by the medicine.

The ſolution was now omitted, and he took, on the 9th, four grains of calomel. This child had no relapſe, and has continued ſince to enjoy enjoy good health, although the tumour in the fide did not wholly difappear till the beginning of the year 1793.

### CASE II.

October 4.—Hannah Peters, a black, aged thirty-fix years, has been for two months paft affected with an intermittent fever; at prefent a paroxyfm comes on every day at noon. During the hot fit, fhe has a confiderable pain of the head, efpecially over the eyes, which continues till evening, and is gradually abated by the fweat which then breaks out. Her ftrength and appetite are much diminifhed.

Capiat folutionis mineralis guttas x. ter die.

6. Had no return of fever yefterday at the ufual time; but towards evening had a flight cold fit, fucceeded by heat and fweating. The paroxyfm, however, was neither fo fevere, nor of fo long continuance as ufual. She felt a little griping in her bowels.

Repetatur Solutio.

8. Has omitted the folution two days, and has had a return of the hot fit each day at the ufual time, without the preceding cold ftage. She was defired to continue the drops regularly.

16. Has

16. Has taken the ſolution regularly ſince the laſt report, during which time ſhe has, not had the leaſt return of her ague, nor any pain of the bowels.

Omittatur ſolutio et capiat Infus. Cort. Anguſt. ℥iij ter die.

### CASE III.

October 10.—David Edmonds, a black, aged forty years, has had every day, for near a month paſt, a paroxyſm of ague, attended with a very ſevere pain of the head. Of late the fit has only returned every ſecond day, beginning about one o'clock, P. M. In the abſence of the paroxyſm he has no complaint but languor and debility.

Capiat ſolut. min. guttas x. ter die.

11. Had a ſlight attack yeſterday evening, which did not continue long; he felt no griping or nauſea from the ſolution.

Repetatur Solutio.

16. Has neglected his medicine for ſome days, during which he has miſſed the cold fit, but had a pretty ſmart hot fit every day, towards evening.

Repetatur Solutio.

20. Has had no return of the cold or hot fit

ſince

ſince the 16th: he continues the ſolution without experiencing any diſagreeable effect from it.

### CASE IV.

Octob. 5.—J. Barnes, aged thirty-ſix years, of a fair complexion, and florid, with red hair, was attacked with the remittent fever about the end of Auguſt laſt, from which he recovered by a liberal uſe of the bark; but ſoon after, on returning to work, and expoſing himſelf too much in the ſun, he ſuffered a ſevere relapſe in the beginning of September. His complaint, however, yielded again to the bark, but left him greatly enfeebled. During the remainder of the month of September, he continued to take from ℥i to ℥iſs of bark every day, and returned to his work. About a week afterwards he was ſuddenly ſeized with a cold fit, followed by a hot ſtage and a profuſe perſpiration, which left him very weak during the apyrexia. His pulſe is now 100, rather hard and quick: he has a ſevere attack every day at noon, attended with vomiting, and, during the hot fit, with a quick and hurried reſpiration; he is hot and and restless till late in the evening, and has then very profuse night sweats.

Capiat solut. min. guttas x. ter die.

Oct. 6. The solution did not disagree with him. He had a slight return of the paroxysm yesterday.

Repetatur Solutio.

8. Has had no return of the fit, nor felt any sensible effect from the medicine. He perspired much at night; has great debility and languor, with little appetite.

10. The symptoms are nearly as before; he did not rest well, but had no return of the paroxysm,

Capiat opii gr. ij h. s. Repetatur Solutio.

12 The solution was yesterday omitted; he rested better with the pill: in other respects finds no alteration.

13. Had a return of the paroxysm yesterday; the cold stage lasted half an hour, the hot stage about two hours. He was much relieved by the opium, and sweated very profusely after it.

14. Had another slight fit yesterday evening, the cold stage being very short; he sweated much: does not recover his strength or appetite. As he could not be prevailed upon to take the bark again, I directed that four ounces of the following following infuſion ſhould be taken three times a day:

℞. Corticis Anguſturæ ℥j Cremor. Tart. ʒij Aquæ pur. ℔iſs.

By this plan his appetite became better, and he regained his ſtrength in ſome degree; but in a week or ten days he relapſed into his former ſtate, having every day an ague fit, which was, however, relieved by two grains of opium, taken at the commencement of the cold ſtage. He now began to take the bark to the amount of ℥iſs a day, which finally put a ſtop to the ague; notwithſtanding, he recovered his ſtrength ſo ſlowly, that it was thought neceſſary, ſix weeks afterward, to ſend him to England for the effectual reſtoration of his health.

### CASE V.

October 14.—A. Richardſon, a black, aged forty years, ſince her recovery from a remittent fever in Auguſt laſt, has continued in a very debilitated ſtate, and for ſome time paſt has been affected with an intermittent fever, the cold fit of which comes on daily at four o'clock, P. M. is very ſevere, and of long duration.

Much

Much pain of the head, and frequent vomiting attend the hot fit, which continues the greateſt part of the night, and is ſucceeded towards morning by a ſlight partial ſweat: ſhe remains very weak till the commencement of the next paroxyſm; her appetite is much impaired; her body open.

Capiat ſolut. min. guttas x. ter die.

16. Has taken the ſolution two days, and has had no appearance of the ague, except a little uneaſineſs and yawning about the time of its uſual attack. No ſenſible effect is produced by the medicine.

Repetatur Solutio.

17. Had a return of the paroxyſm yeſterday; the cold fit was ſhort, but ſevere; the hot fit was alſo violent, and terminated by a profuſe perſpiration; after which, however, ſhe appeared more eaſy and compoſed than uſual. She complained of no griping or nauſea from the medicine.

Repetatur Solutio.

24. Has had no return of the paroxyſm ſince the 17th, nor any ſymptoms of its approach. She continues ſtill very weak, and has little appetite.

Omittatur ſolut. Capiat infuſ. gent. c. ℥ij ter die.

28. Has had no return of the fit. She begins gins to recover her ſtrength and appetite.

Repetatur Infuſ.

### CASE VI.

Nov. 2.—Mary Bowler, aged forty years, a black, has been for ſix weeks affected with a tertian ague; the cold fit is ſevere; the hot fit, which is very violent, and attended with great pain of the head, generally continues all night, and ſometimes part of the next day, without any ſweating ſtage. She is much debilitated, but has a tolerable appetite.

Capiat ſolut. min. guttas x. ter die.

4. Had a return of the paroxyſm yeſterday, after the third doſe of the ſolution. The fit returned at the uſual period, and in the ſame manner as before. No ſenſible effect was produced by the medicine.

Repetatur Solutio.

8. Has had no return of the cold fit ſince the 3d; the hot fit occurred about the uſual time, but it was ſhorter and much leſs ſevere than ordinary.

Repetatur Solutio.

12. Has had no return of the paroxyſm; ſhe complains of a little griping in her bowels, and continues ſtill weak.

Omittatur ſolut. min. Capiat inf. gent. c. ℥ij ter die.

20. She makes no complaint, and has nearly recovered her health and ſpirits.

Repetatur Infus. Gent. c.

### CASE VII.

Nov. 1.—E. Perth, a black, aged forty-five years, has been for near ſix weeks paſt affected with an irregular intermittent, which moſt commonly follows the tertian type. The cold fit is ſevere, and very uncertain in the time of its attack and in its duration. In the hot fit ſhe complains of exceſſive pain of the head, eſpecially over her eyes, and of great pain of the back. The hot ſtage generally continues all night, ſeldom terminating by regular ſweats: it is followed by much laſſitude and uneaſineſs through the enſuing day. Her ſtrength is greatly impaired, her appetite bad; and ſhe is very coſtive.

Capiat ſtatim Sal. cathart. amar. ℥i. Cras incipiat ſumere Sol. min. guttas x. ter die.

6. After taking three doſes of the medicine, ſhe had a return of the paroxyſm on the 3d, but thought the cold fit later in its approach than uſual, and ſhorter. The hot fit continued through a great part of the night, but the pain of the head was much lefs fevere. She has had no return of the paroxyfm fince, and feels only a little griping from the medicine.

℞ Repetatur Solutio.

10. Has had no return of the paroxyfm fince the 3d. She complains only of debility and want of appetite.

Omittatur Solut. Capiat Infus. Gent. c. ℥ij ter die.

14. Begins to recover her ftrength; her appetite is alfo better.

Repetatur Infus. Gent. c.

### CASE VIII.

Octob. 3.—Ann Bowler, a black, aged fourteen years, has been, for fome weeks paft, affected with an irregular tertian, which is fometimes, but not generally, preceded by a cold ftage. The hot ftage continues during the greater part of the day, and feldom terminates by fweating. Her body is open; her appetite much impaired.

Capiat Solut. min. guttas viii: ter die.

10. The folution has now been taken for a week, during which time fhe has had no return of of the ague, nor has felt any nauſea or griping from the medicine. No complaint remains but debility.

### CASE IX.

October 4.—Dinah Lawrence, a black, aged forty-four years, is every other day, about ſix o'clock P. M., ſeized with a ſevere cold fit, followed by great heat and violent pain of the head, eſpecially over the eyes, which ſymptoms continue through the whole night, and are not ſucceeded by any regular ſweating ſtage; ſhe is coſtive, and much debilitated; ſhe has had this complaint near three months.

Capiat ſtatim Sal. cath. am. ℥vi; et cras Solut. min. guttas x. ter die.

10. Has had no return of the fit ſince ſhe began to take the ſolution; ſhe finds no diſagreeable effect from it: is ſtill coſtive.

Repetantur Sal cathart. et Solut. min. ut antea.

14. Feels no complaint but what proceeds from debility; her appetite is better; ſhe was a little griped by the medicine.

Omittatur Solut. min. Capiat Infus. Gent. c. ℥ij ter die.

### CASE X.

Sept. 24.—Jane Armftrong, of a fair complexion, aged thirty years, is feized every day, at eleven o'clock A. M., with a head-ach fo violent as to produce frequent fhrieking and continual moaning. The pain chiefly affects the crown and one fide of the head; it is in general preceded by a cold ftage, though flight, and of fhort duration. The hot fit, which is not very violent, continues till night, when it abates along with the pain; but is not entirely removed till morning: the paroxyfm is ufually terminated by a profufe perfpiration. The patient is naturally of a delicate conftitution, and has of late been much reduced by the remittent fever, from which fhe recovered very flowly.

Capiat Opii gr. iij et Tart. emet. gr. ½ ingruente paroxyfmo.

25. The head-ach was almoft entirely removed within half an hour after taking the pill; the paroxyfm terminated alfo more fpeedily than ufual. Being very coftive, fhe was ordered to take half an ounce of purging falt the following morning.

26. The

26. The falt operated gently; fhe had a very violent return of head-ach at the ufual time, which was relieved by the opium taken alone.

October 4.—She refufes to take the bark: fhe has every day had a return of head-ach at the ufual time, which was however removed by the opium.

Capiat Solut. min. guttas x. ter die; et repetatur Opium fub initium paroxyfmi.

10. Has had no return of the paroxyfm fince fhe began the folution; feels no inconvenience from its ufe, but a flight diarrhœa, without any pain.

Repetatur Solutio.

14. Has had no return of the head-ach; fhe fweats much at night; is very weak, and has no appetite.

Omittatur Solut. Capiat Infus. Cort. Anguft. ℥iij ter die.

This woman has never had a return of the paroxyfm, though a twelvemonth has now elapfed fince the laft report. She gradually recovered her ftrength by the ufe of tonic remedies.

### CASE XI.

Sept. 12.—Jeffe George, a black, aged twenty years, was yefterday afternoon feized with a fevere cold fit of an ague, which continued upwards of two hours, and was fucceeded by great heat, fevere pain of the head, naufea, pains all over his body, more efpecially in the back and loins, great reftlefsnefs, and anxiety. Towards morning a general but not profufe perfpiration took place; the feverity of the head-ach at the fame time abated, and all the other fymptoms wholly difappeared: he has much thirft; his fkin is cool; his pulfe 72, and foft.

Capiat Solut. min. guttas x. ter die.

13. He had a return of the paroxyfm laft night, at eight o'clock, four hours later than the former one. The cold fit, though very fevere, did not continue long; the hot fit was ftrong; the head-ach lefs violent. He had a very profufe perfpiration this morning. His fkin is now cool and moift, and his tongue clean; but fome pain ftill remains over the orbits of the eyes; he complains of thirft, and is coftive.

Capiat Sal. cathart. am. ℥i—Repetatur Solut. min.

14. The head-ach continued yefterday till the afternoon, and then went off; the falts were not taken till this morning. He refted well laft night, and makes no complaint but of debility.

Repetatur Solutio.

15. He

15. He continued free from complaint yesterday, till towards evening, when he became hot and feverish ; and after a very uneasy night, he, this morning, at eight o'clock, had a severe cold fit, attended with violent head-ach, which lasted near an hour. Two grains of opium, taken at this time, brought on a sweat, and terminated the paroxysm.

Repetatur Solutio.

16. He slept well last night, and feels no complaint but from debility. He has omitted the drops this day.

Repetatur cras Solutio.

17. Has had no return of the paroxysm ; he feels no complaint but a slight griping from the solution.

℞. Tinct. Opii et Solut. min. āā ʒij m. capiat guttas xx. ter die.

20. He has had no return of the paroxysm since the 15th. At that time he probably brought it on by having exposed himself the night before to the damp evening air in his shirt. He feels no griping, or sickness, from the drops, which he still takes. He returned to his work this day.

### CASE XII.

Auguft 12, 1793.—Mr. T———, a European, of a dark complexion, with black hair, was fuddenly feized, two days ago, with an acute pain of the head, chiefly over the orbits of the eyes, attended with naufea and vomiting. Thefe fymptoms were foon followed by great heat and reftlefsnefs, which continued through the whole night, and yielded in the morning to a profufe perfpiration. On the 11th he was free from complaint; walked about, and ate heartily. In the evening, however, he was feized with a very fevere fhivering fit, which continued near two hours, and was fucceeded by great heat and reftlefsnefs, by fevere pain above the eyes, and bilious vomiting. He was again relieved in the morning by a copious perfpiration. At ten o'clock, A. M. his fkin was ftill hotter than natural, and his pulfe rather quick; in other refpects he appeared free from complaint.

Capiat Solut. min. guttas x. ter die.

13. The firft dofe of the folution yefterday produced vomiting; the fecond gave him three ftools;

ſtools; the laſt had no particular effect. He paſſed an eaſy night, without feeling any ſymptom of the fit, except a general uneaſineſs, which, however, ſoon went off. He complains this morning of ſlight pain over his forehead.

Repetatur Solutio.

14. The medicine again produced ſickneſs, and a ſlight diarrhœa, though he only took two doſes of it. He remained well till two o'clock, P. M.; he then became very hot, and had a ſevere return of the head-ach, attended with nauſea and vomiting. The heat, pain, and reſtleſsneſs continued till this morning, when a copious perſpiration took place, with which he is yet affected.

At ten o'clock A. M. his pulſe is 130; his ſkin pretty cool; his head-ach almoſt gone; his tongue ſomewhat furred. He complains of thirſt, and of ſlight pain of his bowels, with a ſenſation of numbneſs about the umbilicus

Omittatur Solut. Capiat pulv. Cort. Peruv. ʒi ſecunda quaque hora.

At ſix o'clock, P. M. he has a very ſlight head-ach, with a ſenſe of weight in the forehead; his eyes are more prominent and brighter than uſual. He has taken two doſes of bark ſince noon, the firſt of which produced vomiting; he has had one ſtool to-day; his urine is very high coloured; pulſe 130, ſoft, and leſs quick than in the morning.

Repetatur Cort. et capiat h. s. Tinct. Opii et Vin. Antim. āā guttas xxx.

15th. Ten o'clock, A.M—he has had a good night; ſome pain ſtill remains over his eyes, but it is leſs ſevere; his ſkin is rather hot, but moiſt; pulſe 112; his tongue dry and white; his urine high coloured, with a light cloud ſuſpended in it. He complains much of thirſt and fever, and of a pain in his back. He has taken, ſince yeſterday noon, ℥iſs of Peruvian bark.

Repetantur Cortex, Tinct. Opii, et Vin. Antim.

16. He paſſed an eaſy night, and enjoyed ſome refreſhing ſleep; he complains only of a ſlight pain over his eyes, and is able to ſit up. He had two ſtools in the night; his tongue is cleaner, but ſtill dry; pulſe 104 and ſoft, but eaſily quickened by the leaſt exertion. His urine is not ſo high coloured, and exhibits a flocculent cloud. He took ℥iſs of bark between ten o'clock, A. M. yeſterday, and ſix o'clock this morning.

Repetantur Cortex, Tinct. Opii, et Vin. Antim.

17. He was much griped yeſterday by drinking ſome cyder; has no complaint this morning ing but from weaknefs. His pulfe is 104, and foft; his tongue clean and moift. His urine is much paler than before, and has a kind of gelatinous ftriated cloud fufpended in it.

The fame medicines were repeated.

18. He feems much better in every refpect; his appetite is returning; his pulfe 90, and foft.

He continued the bark a few days longer, and had no return of complaint.

### CASE XIII.

October 4.—Ann and Eliz. Davis, blacks, the former five, the latter fix years old, have been for fome time paft affected with quotidian agues. The cold fit comes on at four o'clock, P. M.; is very fevere, and frequently attended with vomiting. The hot fit ufually continues the whole night, being attended with great reftleffnefs, anxiety, and acute pain over the eyes; but is feldom fucceeded by a regular fweating ftage. Their appetite and ftrength are much impaired.

Capiant Solut. min. guttas vj. ter die.

9. Each of them had a return of the cold fit on on the 4th, after the third dofe of the folution. They have fince had no return.

Repetatur Solutio.

11. There has not been any appearance of the paroxyfm, nor any difagreeable effect from the medicine.

### CASE XIV.

John Oliver, a black, aged five years, who was affected nearly in the fame manner as the two laft patients, began, on the 16th of Auguft, to take four drops of the folution three times a day.

23. He had a return of the fit on the 16th, 17th, and 18th, but it commenced every day later, was lefs fevere, and of fhorter duration. Since the 18th he has had no fit, although the folution was difcontinued. A flight tumefaction of the face has been obferved for two days paft, but is at prefent fubfiding. He felt no naufea or pain from the medicine.

### CASE XV.

Dec. 10.—Mary Jones, a black, aged thirty-six years, about three months ago was affected with a remittent fever, from which ſhe recovered very ſlowly, and has ſince continued in a ſtate of great debility. She has of late been ſubject to violent pains in the bowels, attended with diarrhœa. During the laſt month ſhe has had a regular tertian ague, the cold fit of which begins generally at ſun-ſet, but is not very ſevere, nor of long continuance. The hot fit is long and ſevere, being attended with violent head-ach, intenſe thirſt, and great reſtleſſneſs. Theſe ſymptoms are not terminated by a regular ſweating ſtage; and have often no remiſſion till the middle of the following day. She is feeble, and much emaciated.

Capiat Solut. min. guttas x. ter die; et Opii. gr. ij. ſub acceſſionem paroxyſmi.

12. The hot fit was much relieved by the opium; the paroxyſm was ſhorter, and the head-ach leſs ſevere. She is very coſtive.

Repetatur Solut. min. et capiat Sal. cathart. ℥ſs mane.

15. Con-

15. Continues the ſolution without feeling any ſenſible effect from it. She has had no cold fit or head-ach during the two laſt paroxyſms. The hot fit was much leſs violent and of ſhorter duration than formerly.

Repetatur Solutio.

18. Has had no return of the fit, nor any appearance of it ſince the laſt report; nor does ſhe perceive any nauſea or griping from the ſolution. Her appetite is ſtill much impaired.

Repetatur Solutio. Capiat Inf. Cort. Anguſt. ℥ij ter die.

22. There has been no return of the paroxyſm. She finds her ſtrength and appetite much increaſed by the infuſion.

The uſe of the ſolution was diſcontinued.

### CASE XVI.

Feb. 1.—John Jones, a European, of a ſallow complexion, aged twenty-eight years, is affected in the afternoon, every other day, with a violent cold fit, attended with rigors, and ſucceeded by a regular hot fit and ſweating. Until within a few days, he has been able to do his duty on ſhip-board as a ſeaman; but the paroxyſm returns now with ſo much violence, as to confine him to his hammock. He has taken a large quantity of Peruvian bark at different times, which has never failed to prevent the next return of the paroxyfm; he has always, however, had a relapfe in a few days, through intemperance, and expofure to the night air.

Capiat Solut. min. guttas x. ter die.

8. Has taken the folution without perceiving any fenfible effect from it. The paroxyfm returns as ufual, but, as he fays, with much lefs violence.

Repetatur Solutio.

15. The paroxyfm returns as ufual, but is fhorter and lefs fevere. Through miftake, he has taken the folution only before the attack of each paroxyfm.

Repetatur Solutio; et capiat guttas x. ter die.

20. He has had no return of the paroxyfm fince he took the folution as directed, and feels no naufea or griping from it.

He continued the medicine a few days longer, and was reftored to perfect health.

CASE

### CASE XVII.

Feb. 1.—Ann Wicks, a mulatto, aged forty years, has been for a month paſt affected, every other day, with a violent cold fit, attended with rigors, and ſucceeded by great heat. She has alſo a ſevere pain over the forehead, and on one ſide of the head, extending to the neck and ſhoulder of the ſame ſide. There is much ſtiffneſs and pain in moving the neck during the intermiſſion. The cold ſtage commences about five o'clock, P. M. and continues near an hour. The hot fit does not terminate before morning, and is ſeldom ſucceeded by a regular ſweating ſtage. She is much debilitated by the long continuance of the complaint, and has lately given ſuck to a young child. Her appetite is alſo greatly impaired.

Capiat Solut. min. guttas viij ter die.

4. She has had no return of the cold fit. The hot fit continued only part of the night, and was unattended with head-ach or any other diſtreſſing ſymptom.

Repetatur Solutio.

12. She

12. She has had no return of the paroxyfm, and feels no ill effects from the folution. Her ftrength is fomewhat increafed, but her appetite is ftill bad.

Omittatur Solutio. Capiat Inf. Gent. c. ℥ifs bis die.

### CASE XVIII.

Mrs. D. a delicate woman, of a fair complexion, aged twenty-four years, in the month of Auguft laft had a mifcarriage, from which fhe recovered without much trouble, and enjoyed a tolerable ftate of health till the beginning of October, when fhe was feized with the common remittent fever of the place. From this complaint fhe alfo recovered within a fortnight, by taking largely of the bark in powder and decoction. About the end of the month, however, fhe fuffered a relapfe, and made a very flow progrefs towards recovery; her ftomach being only able to retain the bark in the form of a decoction. She laboured under great debility, very profufe night fweats, and frequent hectic flufhings during the day, with lofs of appetite, and general tremors on ufing the leaft exercife. Thefe fymptoms were at length confiderably alleviated by the infufion of Anguftura bark, elixir of vitriol, and other tonics.

Dec. 15. Yefterday, at fix o'clock, P.M. fhe had a cold fit, with rigors, which lafted near half an hour, and was fucceeded by a hot fit, attended with great pain of the head, naufea, vomiting, and reftleffnefs, which continued through the whole night; towards morning fhe was relieved by a partial fweat, but remained very weak and languid.

16. Yefterday, at the fame hour, fhe had a return of the paroxyfm, the fymptoms of which were mitigated by an opiate taken foon after its commencement: fhe had a copious perfpiration during the night, and feems free from complaint this morning.

18. Had a return of the fit on the 16th and 17th, but was relieved as before by an opiate. She refufes to take bark.

Capiat Solut. min. guttas viij. ter die.

20. She has had no return of the cold fit fince the 18th. The hot fit was much fhorter and lefs fevere. She experiences no inconvenience from the medicine.

Repetatur Solutio.

22. She has had no return of the paroxyfm, but feels a flight pain in her bowels.

Capiat ftatim Tinct. Opii guttas xx. et fp. lav. c. ʒfs.

Repetatur Solut. min.

24. The

24. The pain of her bowels was removed by the opiate; ſhe has had no return of the paroxyſm; reſts pretty well during the night, but ſweats much towards morning.

Omittatur Solutio; et capiat Infuſ. Gent. c. ℥i ter die.

30. Her ſtrength is returning. Her appetite is good, and ſhe has had no return of the paroxyſm.

This lady continued to enjoy a good ſtate of health, till the 20th of March, 1793, when ſhe was affected with a diarrhœa, attended with acute pain in her bowels, chiefly about the umbilicus. She was ſoon relieved from theſe complaints by an opiate, and a few powders, conſiſting of the colombo root joined to an aromatic: but on the 25th, ſhe had a return of an intermittent fever, the cold fit of which was very ſevere. It began at ſix o'clock in the evening, continued near two hours, and was followed by a hot fit, which laſted all night, terminating towards the morning in a ſlight perſpiration, and leaving her low and weak the remainder of the day.

28. She refuſed yeſterday to take an opiate on the approach of the cold fit, having on former occaſions found her head diſagreeably affected by it. The paroxyſm proved very ſevere: the hot fit continued all night, and was ſucceeded by partial ſweats about the head and neck. She is very weak this morning, and complains of a great pain of the head and back; of lowneſs of ſpirits and general uneaſineſs.

Capiat Solut. min. guttas viij. ter die, ex Infuſ. Cort. Anguſtur. cyatho.

30. The ſolution did not diſagree with her in any reſpect; ſhe had a cold fit laſt night, but it was much leſs ſevere than uſual: ſhe is alſo in better ſpirits to-day.

Repetatur Solutio.

April 1. There was no cold fit yeſterday; but ſhe had a hot fit, which continued all night, and terminated in a very profuſe perſpiration. Her ſpirits are much revived; ſhe is conſiderably ſtronger, and has a better appetite.

Repetatur Solutio.

6. She continues the ſolution without feeling any inconvenience from it; and has had no return of the fit, or night-ſweats, ſince the 1ſt: her appetite at preſent is good.

Repetatur Solutio.

8. She has had no fit, and recovers her ſtrength gradually. No nauſea or griping has ever been produced by the ſolution.

Omittatur Solutio. Capiat pulv. rad. colomb. gr. xv. ter die.

CASE

### CASE XIX.

Feb. 1, 1793—Mrs. H. of a fair complexion, aged twenty-four years, during the months of September and October laſt, had two ſeveral attacks of the remittent fever, from which ſhe recovered ſpeedily by means of the bark: ſince that time ſhe has continued in a very weak irritable ſtate, ſubject to pains of the bowels, and to frequent though ſlight returns of a febrile complaint, which continued only for a day or two, and commonly yielded to an opiate. On the 27th of January ſhe had a cold fit at eight o'clock in the morning; this was ſucceeded, in about an hour, by a burning heat of the ſkin, with fluſhing of the face, great reſtleſſneſs, and ſevere pain of the forehead. Her eyes, at the ſame time, appeared bright and prominent; ſhe complained alſo of a ſenſe of heat in them, and was unable to bear the light. In the evening, a copious perſpiration enſued, and conſiderably alleviated the ſymptoms; ſhe had, however, a ſlight head-ach through the whole night: the fit has returned every morning at the ſame time for the laſt four days.

Feb. 2. The paroxyſm appeared this morning as uſual, with a ſevere cold fit and head-ach, but was rendered much ſhorter and leſs diſtreſſing by an opiate draught taken ſoon after its acceſſion.

Capiat Solut. min. guttas viij. ter die.

5. She had a ſlight return of the cold fit this morning, with a little head-ach, but the paroxyſm was of ſhort duration.

Repetatur Solutio.

6. She has had no cold fit to-day, nor any pain of the head; the hot fit returned at the uſual time. Her face is much fluſhed, and her ſkin hot, but with leſs anxiety and reſtleſſneſs than heretofore: ſhe finds no inconvenience from the ſolution. The opiate was not taken to-day.

Repetatur Solutio.

10. She has had no return of the paroxyſm, nor has felt the ſlighteſt ſymptom of its approach ſince the 6th; ſhe complains only of a ſlight pain or uneaſineſs in her ſtomach. Her appetite ſtill continues weak.

Omittatur Solut. min. Capiat tinct. opii guttas xx. ſtatim.

14. She begins to recover her ſtrength and appetite; appetite; the pain of the ftomach was immediately removed by the opiate.

All the patients whofe cafes are here related, have continued to enjoy good health fince cured by the folution; and though feveral months have now elapfed, none of them have experienced the leaft unpleafant fymptom which could be attributed to that remedy. The women continued to labour under a fuppreffion of the catamenia, until their ftrength was entirely reftored.

Mrs. H. (Cafe XIX.) though enjoying a good ftate of health, had no appearance of them till the middle of Auguft laft, when they flowed for feveral days rather profufely.

In Cafe IV. I had little profpect of fuccefs from the ufe of the folution, the child having become very weak and irritable by frequent relapfes: but as he had for a length of time taken the bark in large dofes without any effect, I was induced to try the mineral folution, with a view of checking the returns of the paroxyfm, hoping afterwards to complete the cure by the bark; which might prove more effectual after its ufe had been fufpended a few days.

In Cafes I. X. XIII. and XIV. there was an evident enlargement of the fpleen, forming a projection below the cartilages of the ribs. In Cafe X. it was fo large as to extend nearly as low as the crifta of the os ilium. After the ague had ceafed, the patient continued to ufe corroborant medicines, taking at the fame time fmall dofes of calomel, but without any fenfible effect on the tumor; it yet remains nearly in the fame ftate, not, however, caufing much uneafinefs. In Cafes XIII. and XIV. as the patients fpeedily regained their health after the ague had ceafed, and felt no uneafinefs from the enlargement of the fpleen, I did not think it proper to ufe any medicine, excepting a purgative dofe of calomel occafionally, becaufe, in many fimilar cafes, where this medicine had been ufed, even in very fmall dofes, a falivation was very foon excited, the tumor not being at all affected by it, whereas the patient was rendered extremely weak and irritable. The only inftance of tumefaction which could with any probability be referred to the ufe of the folution, was Cafe XIV. in which, however, it proved fo flight, as fcarcely to deferve notice.

In order to give the mineral folution a fairer trial, I avoided, in many inftances, making ufe ufe of two very powerful means ufually employed for the purpofe of diminifhing the violence of the paroxyfm, and which frequently indeed put a total ftop to it; I mean, opium and emetics: when two grains of opium are given a fhort time before the paroxyfm is expected, it feldom fails to bring the fit to a fpeedy termination by a profufe fweat; and generally relieves the violent pain of the head, which is fo diftreffing during the hot fit, as in Cafes X. and XV. The recurrence of the paroxyfm being once obviated, I have found that a full dofe of opium at night affords more comfortable reft, and more certainly prevents the folution from affecting the bowels, than when the tincture of it is added to the mineral folution; a mixture of this kind always becomes turbid, and the opium is partly feparated.

Intermittents partake much of the nature of remittents, and the two difeafes have a very uncertain boundary; whenever, therefore, the intermiffions are imperfect and indiftinct, the exhibition of an emetic is attended with moft beneficial effects. In many inftances this practice puts a temporary ftop to the returns of the fit, and in every cafe confiderably diminifhes its violence. The proper time of giving an emetic emetic, is about two hours before the paroxyſm is expected; and the beſt mode is to employ a ſolution of tartarized antimony in divided doſes, at intervals of eight or ten minutes, until full vomiting be produced. When the patient has vomited a few times, and his ſtomach is a little ſettled, a more moderate doſe of the antimonial ſolution, joined to a full doſe of opium, ſeldom fails to produce a copious perſpiration before the attack of the cold fit. This method generally ſucceeds in preventing the immediate recurrence of the paroxyſm: but in thoſe caſes where the intermittent has continued long, and ſeems to return by the power of habit, it will be proper to repeat the emetic once or twice more before the time when the paroxyſms are expected.

I think it proper here to obſerve, that antimonials, in the nauſeating doſes in which they are frequently given during the remiſſion or apyrexia, with a view of procuring a more perfect ſolution of the diſeaſe, are ſeldom found adequate to the purpoſe; on the contrary, the continued action of ſo powerful a ſtimulus, in general, produces a correſpondent ſtate of debility, and relaxes the muſcular fibres of the ſtomach ſo much, that neither food nor medicine can be properly retained.

The remittent fever is, in many caſes, very mild; whence the remiſſion has often been miſtaken for an intermiſſion. This miſtake is more liable to be made when the remittent fever is preceded by an evident and ſevere cold ſtage at each return of the paroxyſm, and is followed by a regular hot, and ſweating ſtage. The remittent may, however, be diſtinguiſhed from the intermittent fever; 1ſt, by a ſlight pain which remains fixed in the forehead, or over the orbits of the eyes, during the apyrexia; 2dly, by the pulſe, which, though not more frequent than in health, yet retains a degree of quickneſs or ſharpneſs through the whole of the remiſſion; 3dly, by the ſtate of the ſkin, which, though moiſt, feels hotter than natural. In ſuch caſes I have not found the mineral ſolution ſo ſucceſsful as in thoſe where the intermiſſion was complete; for which reaſon it ſeems moſt prudent to place our ſole dependance upon the bark, as in Caſes IV. and XII. Sometimes, however, when the patient could not be prevailed upon to take the bark in proper doſes, I have found much advantage from joining it with the mineral ſolution, by which means a ſmaller quantity tity of bark will anfwer the intended purpofe. But whenever immediate danger prefents itfelf, or is to be apprehended from a continuance of the fever, the bark, given in large dofes, is the only medicine to be depended on.

The mineral folution ufually fails in fome irregular cafes, which at firft view refemble intermittents, and have been improperly ranked with them, under the denomination of erratic or anomalous intermittents. A morbid increafe of irritability appears to be the foundation of thefe irregular complaints; they affect principally thofe who have been debilitated by frequent attacks of fever, or by lingering difeafes; alfo children; and women, more efpecially thofe who give fuck; and, in general, perfons of a weak delicate habit. The fymptoms which occur in thefe complaints are nearly as follow: during the afternoon, or towards evening, the patient becomes uneafy and reftlefs; his fkin feels dry, and is hotter than ufual, but without imparting the burning heat ufually obferved in the hot ftage of intermittents; the pulfe becomes quick, and rather more frequent than natural; a pain is fometimes felt in the head, either on the crown, or on the back part of it; the thirft is feldom very great; difagreeable clammiclamminefs, however, takes place in the mouth. Thefe fymptoms are fometimes preceded by flight chills running down the back, which, however, when they do occur, are not of long continuance, and never accompanied with violent fhiverings.

In this manner the patient is harraffed during the whole night*, but obtains relief towards morning, when a partial fweat fometimes appears about the head and breaft. Excepting a degree of languor and debility, little or no complaint is felt till the return of evening. The duration of thefe complaints is very uncertain; they fometimes affect the patient daily for one or more weeks; at other times abate or difappear for a few days, and then return as before. Whatever increafes the irritability of the body, may be confidered as an occafional caufe of them; but the moft common as well as moft powerful one is too much fatigue, along with expofure to a hot fun.

In thefe cafes, after evacuating the ftomach and bowels by a gentle emetic or purgative, it is commonly fufficient to exhibit fome tonic, in a form agreeable to the patient's ftomach. The

* Hence the denomination of night-fever.

Peruvian bark does not appear to produce any better effects than the other vegetable tonics, as Gentian, Colombo, &c. An infusion of Angustura bark is what I most frequently employ, and find most useful, taking care to prevent the costiveness arising from its use, by giving, at proper intervals, a dose of calomel.

For children, who cannot easily be induced to take bitters, after the previous use of an emetic, a few moderate doses of calomel are commonly sufficient.

Notwithstanding the effects of arsenic appear to be equally as powerful and nearly as certain as those of bark in the cure of intermittent fevers, yet it must be confessed that perfect strength is less speedily recovered when the cure has been accomplished by arsenic alone, than when bark has been employed. This objection to the use of arsenic is of less consequence in cold climates, where, if the ague has not been of long standing, the debility induced by it is seldom very considerable. In tropical countries, however, a few attacks of an intermittent frequently reduce the patient so much, that even when the paroxysm has ceased to return, the extreme debility which remains, is of of itfelf fufficiently alarming to demand every attention from the practitioner.

It does not appear improbable that the bark owes its fpecific power, in the cure of remittent and intermittent fevers, to fome peculiar principle in its compofition, which has hitherto eluded the refearches of experimenters, and which they have in vain attempted to imitate by various combinations of bitters and aftringents. In whatever this peculiar power of the bark may confift, the fame quality appears to be poffeffed by the arfenic in a confiderable degree. Both remedies probably effect the cure of intermittents, by their action upon the fibres of the ftomach, fince they often operate fpeedily, and even in a fmall dofe; but the power of the arfenic feems to ceafe here; whereas the bark is capable of reftoring tone to the fyftem in general. The fame effect may perhaps be nearly obtained by joining fome tonic medicine to the arfenic. With this view, in many cafes, after the folution had been taken a week or ten days, I difcontinued its ufe, and ordered the patients to take the Infuf. Anguft. Infuf. Gent. c. &c. until their ftrength was completely reftored. It may be found ftill more advantageous to employ ploy thefe remedies along with the mineral fo-lution.

Arfenic feems to have been oftener employed as a medicine in Germany, than in any other part of Europe; but chiefly by the empirical clafs of practitioners, which no doubt prevented its introduction into general ufe. Many eminent phyficians in Germany, as well as elfewhere, have, however, fpoken highly in its favour, and occafionally prefcribed it. Like many other active remedies, it has been much abufed by the bold and the ignorant, and has been given in dofes which no man of prudence would venture to direct; efpecially as we know that the fame good effects may be obtained by moderate dofes of it, and without the leaft rifk. The following obfervations, extracted from a German work *, will fhow how extenfively this medicine has been ufed on the Continent, and how little caution has been obferved in its exhibition.

Dr. Slevogt, Profeffor of Anatomy at Jena, in 1700, recommended the ufe of arfenic, extolling it as the beft, moft certain, and fafeft cure of intermittents, efpecially of tertians and quartans. He employed it in dofes of a grain

* Nicolai Recepten und Kurarten. 8vo, Jena, 1780.

or or a grain and a half mixed with a proper quantity of Theriaca; not only giving it on the days of the apyrexia, but alfo a fhort time before the acceffion of each paroxyfm. He afferts, that in fifty inftances, two or three dofes were fufficient to put a total ftop to the difeafe, and that he never obferved the leaft ill effect from it.

Melchior Friccius* recommends arfenic in intermittents, and declares he has ufed many drachms of it in the cure of fuch fevers; but confeffes that he had often met with relapfes afterwards.

Lanzonus † quotes a letter from Valifnieri to one of his friends, written in 1707, in which he fays the French furgeons were accuftomed to cure long-continued intermittents with a fmall quantity of arfenic: and he adds, that their remedy feemed to refemble much the famous aqua del petefino, which was a ftrong folution of arfenic boiled in a copper veffel ‡.

* De Virtute Venenorum medica. 8vo. Ulmæ, 1701.—See alfo London Medical Journal, Vol. VII. p. 194.

† Lanzoni Oper. omn. med. phyf. 4to. Lauf. 1738. Tom. I. p. 68.

‡ The *Aqua della Toffanina* (fo called from the inventor), *Aquetta di Napoli*, *Poudre de Succeffion*, *Eau Mirable*, &c. were preparations of arfenic frequently ufed as poifons during the laft century.

Keil* praiſes arſenic as a certain and ſafe ſpecific in intermittents, when prepared and adminiſtered in the following manner: half an ounce of white arſenic, finely powdered, is to be put into a glaſs, or tea-cup; half an ounce of diſtilled vinegar is then to be added, and evaporated over the fire, being conſtantly ſtirred at the ſame time with a wooden ſpatula; the ſame quantity of vinegar is again to be added and evaporated in like manner. After this proceſs has been repeated ſix times, the reſiduum is finally to be waſhed with warm water, and dried; a drachm of the dry powder is to be made up into ſixty pills by means of a ſcruple of wafers ſoftened with water. Previouſly to the uſe of the pills, the patient is to take an emetic compoſed of tart. emet. or ſulph. aurat. antim. and a little vitriolated tartar, or ſome purgative medicine on the morning free from fever: the next day, or only a few hours before the acceſſion of the paroxyſm, one of the pills is to be taken faſting, and nothing is to be eaten or drank after it for three or four hours. When this has been repeated three days, during the apyrexia, or a few hours before the

* Anatom. Chirurg. Medicin. Handbuchlein. 8vo. Konigſberg, 1761.

attack of the paroxyſm, the fever commonly ceaſes. He affirms that this practice has been attended with ſucceſs in ſeveral hundred caſes, when every other remedy had been employed in vain; that he has never obſerved the leaſt ill effect to accrue from it; but, on the contrary, that thoſe who had before looked thin and ill, had become, in conſequence of it, fat and ſtrong; and that he knew many perſons who had uſed this remedy fifteen or twenty years before, and who continued to enjoy a ſtate of perfect health.

Dr. Jacobi * recommends the uſe of arſenic ſtrongly in fevers: he directs one part of arſenic, and twelve of ſalt of tartar, to be mixed with 180 parts of water, and boiled till one half has evaporated; when cold, as much freſh water is to be added to it as has been loſt by the evaporation, together with a little ſpirit of wine. The doſe for adults is twenty-five drops, to be given on the day which is free from fever, at ſeven A. M., at three, ſix, and nine, P. M. Before the uſe of this medicine, the primæ viæ muſt be evacuated by emetics and purgatives; and the common febri-

* De prudenti uſu Arſenici, ſale Alcalico domiti, interno ſalutari, Diſſert.—Vide Act. Acad. Elect. Mogunt. Tom. I. p. 216. 8vo. Erford. 1751.

fuge remedies ſhould be uſed for ſome time. Dr. Jacobi obſerves that he has employed the above preparation not only in intermittents, but alſo in continued fevers, with the greateſt ſucceſs, and without ever experiencing any bad effects from it.

Heuermann * ſays that arſenic is uſed in Holſtein, at Copenhagen, and ſome other places, as the moſt certain remedy for the cure of intermittents; that he has himſelf given it with conſtant ſucceſs, in fevers, to patients who were not able to retain other medicines on the ſtomach in a proper quantity; and that two caſes, wherein frequent relapſes had occurred, were entirely cured by this remedy. He prepares a ſolution of arſenic in the following manner: half an ounce of white arſenic, and ſix ounces of alkaline ſalt, are added to ℔iſs of water, and then evaporated to dryneſs. The ſame quantity of water is added a ſecond time to the reſiduum, and evaporated to one half, which is coloured red by a few poppies. Of this he directs from ſeven to ten drops to be taken during the day, beginning immediately after the paroxyſm is over, and omitting it a ſhort time before the return of the next. If the

* Vermiſchte Bemerkungen und Unterſuchungen. Vol. I. 8vo. Copenhagen, 1765.

folution produces vomiting, it is too ftrong, and muft be diluted; only one dofe is to be given in twenty-four hours, and the patient muft be kept moderately warm, to promote a gentle perfpiration. Expofure to cold, he fays, is as hurtful during the ufe of this as of other febrifuge remedies, as it difturbs Nature in her operations, and retains in the body the noxious matters which fhe is endeavouring to expel. If in the firft three or four days after the ufe of thefe drops, the fever does not ceafe, he recommends that the fame dofe fhould be repeated twice a day, which commonly proves fufficient. The ill confequences which have been obferved after the ufe of arfenic, as palfy, trembling of the limbs, blindnefs, deafnefs, &c. he afcribes to the improper preparation and imprudent ufe of it; afferting, that it is a fafe remedy when properly prepared.

In the Ephemerid. Acad. Nat. Curiof.* arfenic is alfo celebrated as an infallible fpecific for intermittents. Three or four grains of powdered white arfenic are directed to be put into a fmall uncovered glafs with a proper quantity of water, and placed upon the fire till a folution takes place, when it is to be well ftirred up and given to the patient: the fever, we are in-

* Dec. II. Ann. III. p. 132.

formed, is by this means certainly prevented from returning. The patient ſhould eat nothing for twelve hours before; but a quarter of an hour after having taken the medicine, he is allowed a gill of warm water, in which a quantity of butter is diſſolved, together with the yolk of an egg; after which, nothing more is to be given for ſome hours. There generally follows a conſiderable degree of uneaſineſs, and a profuſe ſweat; and by theſe means, it is ſaid, every intermittent, even a quartan, may be readily cured. Two other formulæ are given in the ſame work*, and recommended as highly uſeful in the cure of intermittents, viz.

℞. Tart. crud. ʒi. Arſen. cryſt. ʒſs. Pip. long. ℥ſs. Lap. prunell. ʒiſs. Specif. febrifug. Crollii ʒiij. M.

The doſe is from gr. v. to ℈ſs.

The other is

℞. Arſen. alb. gr. v. Lap. prunell. vel Nitri depur. gr. xv. M. pro una doſi.

Profeſſor Ackermann † relates, that in Pauſa, a town of Saxony, a ſurgeon's family had been poſſeſſed for more than a century of a ſecret remedy againſt melancholy, which conſiſted of two grains of arſenic mixed with a drachm or more of white ſugar, to be taken

* Dec. II. Ann. V. p. 474.

† Neues Magazin für Aerzte. Vol. II. p. 401. 8vo. Leipſic, 1780.

early early in the morning, along with a large quantity of mucilaginous drink. The medicine produced a violent vomiting, ſo as to agitate the whole body, which continued not leſs than ſix hours; after this, he obſerves, the patient uſually enjoyed a quiet ſleep, and became more rational. The remedy was perſiſted in, care being taken that the effects of the firſt doſe ſhould be completely over before a ſecond one was adminiſtered. Many repetitions of the medicine were not however requiſite, as the diſeaſe, in general, ſoon yields to this mode of treatment; the patient was afterwards directed to continue a mucilaginous diet for a few weeks. Profeſſor Ackermann examined ſome of the patients who had been cured by the ſurgeon at Pauſa, and found that no ill effects had ariſen in conſequence of it. The ſame perſon, it is added, employed arſenic very frequently for the cure of intermittents; he diſſolved two grains of arſenic in a pint of water, and gave two, or three table ſpoonfuls for a doſe every day; under this treatment the fever ſeldom recurred more than twice; but he remarked that the patients were longer in recovering their ſtrength than when the bark had been uſed.

Profeſſor Ackermann farther obſerves, that another ſurgeon in the ſame place likewiſe employed arſenic with great ſucceſs; he gave fifteen drops of a ſolution of arſenic in water, along with alkaline ſalt, but the Profeſſor had not been able to aſcertain the exact proportions. A doſe was ordered to be taken as ſoon as the patient felt the approach of the fit, and a quantity of warm tea was to be drank immediately afterwards. This produced a vomiting, which was encouraged as much as poſſible by repeated draughts of the tea. In this manner, it ſeems, he had cured many obſtinate agues by two or three doſes of the ſolution; and, amongſt others, a quartan which had continued upwards of two years.

From ſome of the foregoing narratives, arſenic ſeems to have been uſed with as little precaution as emetic tartar; and ſince it appears, on good authority, not to have been productive of bad conſequences, even in very large doſes, we may be induced to lay aſide that extreme anxiety with which we generally preſcribe it; and may be encouraged to perſiſt in the uſe of a remedy which, when prudently adminiſtered, is both ſafe and efficacious.

Many of our moſt active and approved medicines, as preparations of mercury and antimony, the ſquill, foxglove, &c., are capable of producing as violent effects in the conftitution, when given in too large a dofe, as arfenic itfelf. All thefe medicines met with the fame, if not ftronger, oppofition when firft introduced, as arfenic does from many at prefent. It is well known that antimonial preparations were declared to be poifonous, and that the ufe of them was prohibited by a decree of the faculty of Phyfic at Paris in the year 1566; which decree was not repealed till 1637. We fhall ceafe, however, to wonder at the prejudices formerly entertained againft thefe medicines, when we confider, that even at the prefent day fimilar objections are made upon the Continent, efpecially in Germany, to the ufe of the bark, a remedy, the reputation of which has been fo fully eftablifhed by the united teftimony of fo many eminent practitioners, fupported by almoft innumerable experiments.

Mr. Theden, one of the moft celebrated furgeons in Germany, and Surgeon General to the Pruffian army, in fpeaking of the treatment of intermittents, obferves*, that when his patients had previoufly enjoyed a healthy ftate of body, he was generally able to effect a cure in fix or

* Unterricht für die Unterwundärzte. 8vo. Berlin, 1793.

eight eight weeks. As he entertained the common idea that bark is apt to produce obſtructions and enlargement of the viſcera, œdematous ſwellings of the extremities, &c. he cautiouſly avoided giving this remedy until he had tried every other means. During the firſt three weeks he employed different medicines, with a view to looſen the morbific matter, and to render it fit for expulſion from the body; he then gave two ounces of bark, in doſes of half a drachm, every two hours. After an interval of eight days, during which only bitters were preſcribed, he ventured again to exhibit an ounce of the bark, and thus completed a cure. He cautions us againſt the uſe of bark whilſt the face retains a yellow tinge, or whilſt the febrile matter remains in the conſtitution; he confeſſes, at the ſame time, that he has ſeen œdematous ſwellings of the lower extremities after agues where no bark had been employed.

Dr. Vogel* is likewiſe of opinion that many cachectic diſeaſes, particularly obſtructions of the viſcera, dropſy, jaundice, phthiſis, tympanitis, coughs, aſthma, hemicranium, deafneſs, cataract, vertigo, &c. are frequently the con-

* Handbuch der praktiſchen Arzneywiſſenſchaft. 8vo. Stendal, 1781.

ſequences

ſequences of an improper treatment of intermittents; more eſpecially when the cure has been attempted by aſtringents, arſenic, &c. or even by an unſeaſonable exhibition of the Peruvian bark, whilſt the morbific matter ſtill remains in the ſyſtem.

The objections to the uſe of theſe medicines are ſo vague, that they appear to originate from popular prejudice and ill-grounded theories, rather than from any juſt practical deductions; they will therefore have little weight with thoſe who are not contented with bare aſſertions, but make actual obſervation and experience the ſtandards of truth.

Having frequently found the moſt beneficial effects from the mineral ſolution, and having never obſerved any ill conſequences to ariſe from its uſe, I may preſume to recommend a trial of it to ſurgeons practiſing in warm climates, and particularly upon the coaſt of Africa.

The high price of bark may ſometimes prevent ſurgeons of ſhips from laying in, at their own expence, ſuch a ſtock of this valuable medicine as will enable them to employ it freely in every caſe which requires its uſe. For notwithſtanding the frequent complaints of ſeveral reſpectable ſurgeons in the navy, the quantity of of bark allowed by government to ſhips on foreign ſtations, is much too ſmall; and moſt of the merchant ſhips trading to this coaſt are ſtill more inſufficiently provided.

Of the two moſt frequent diſeaſes upon the coaſt of Africa, the remittent and intermittent fever, it is certain that the latter, though leſs rapid in its courſe, and apparently leſs dangerous than the former, yet for the moſt part occaſions that irremediable injury to the conſtitution, which ſo often befalls Europeans trading upon this coaſt. There are few, even of thoſe who are ſaid to be ſeaſoned to the climate by long reſidence, who have not ſuffered ſeverely from repeated attacks of intermittents. This in a great meaſure ariſes from the unhealthy ſituation in which they live for the convenience of trade. They generally fix their reſidence on the banks of ſome river, or narrow creek, whoſe oozy ſhores, ſurrounded by mangroves, and excluded from the wholeſome breezes, are a conſtant ſource of miaſmata and contagion; to this muſt be added the debauched and irregular courſe of life which moſt of them lead. Though ſeaſoned to the climate, as they ſuppoſe, their unhealthy ſallow complexions and emaciated bodies, the frequent hectic fluſhings of the face, face, ſwelled legs, &c. attended with obſtructions and enlargement of the abdominal viſcera, ſufficiently indicate to every obſerver the ſhattered ſtate of their conſtitutions. The ague probably ſtill continues to return once a month or oftener, and harraſſes them a few days, without being much noticed; for the ſeverity of the diſeaſe ſeems to be conſiderably abated by its frequent recurrence, though its bad effects in the end are equally certain. As their appetite during the intermiſſion is frequently keen, and even voracious, they flatter themſelves that the conſtitution is not impaired by frequent returns of the diſeaſe; many alſo are negligent, from a confidence in the popular prejudice, that a cold fit ſhows the abſence of danger.

In theſe caſes, therefore, when the bark cannot be procured, or, as more frequently happens, when the patient has conceived a diſguſt for it, and cannot be prevailed upon to take it in a ſufficient quantity, the mineral ſolution promiſes to be a ſafe and effectual ſubſtitute for it.

During the laſt rainy ſeaſon I have had frequent quent opportunities of exhibiting the mineral ſolution in intermittents with the ſame good effects as in the preceding year. Out of the number of caſes which occurred in the preſent ſeaſon, I have ſelected the two following, as being the only inſtances of quartans I have met with ſince I began to uſe the mineral ſolution.

### CASE XX.

Sept. 11, 1793.—John Thompſon, a mulatto, aged thirty years, was ſeized, about two months ago, with an ague, which returned every ſecond day. After the ſecond paroxyſm he took an emetic, and ſoon after the operation of this, an opiate, which appeared to put a ſtop to the diſeaſe. A month ago he was again ſeized with cold ſhiverings, followed by an increaſe of heat, which terminated by a profuſe ſweat. The fit now returns every fourth day; the cold ſtage of which, commencing about noon, is very ſevere: the hot ſtage continues through the whole night, with violent head-ach, and towards morning is relieved by a profuſe ſweating.

ing. His appetite is pretty good; his body open.

Capiat Vesp. Antim. Tartar. gr. ij. cu. P. Ipecac. ℈j.

Cras incipiat sumere Sol. min. guttas xij. ter die.

20. The emetic operated well. He took the solution regularly for four days, and then omitted it, finding no return of his complaint.

30. He has had no return of the paroxysm, nor has taken any medicine since he left off the solution.

### CASE XXI.

Sept. 8.—Anne Crankepoor, a black, aged twenty-eight years, has every fourth day, at noon, a severe cold fit of the ague, which continues near two hours, and is attended with violent rigors and pains of her bones; these symptoms are followed by a hot stage of long continuance, but which terminates by profuse sweating. She is affected, during the whole paroxysm, with violent pain of the head, stomach, and back, which also continue through the intermission, though with some abatement. She has taken an emetic and two anodyne draughts without any relief; and has had no ftool for eight days. Her head-ach is at prefent very fevere; her pulfe quick and frequent; her fkin hot and dry.

Capiat ftatim Camphor. gr. x. Tinct. Opii, guttas xxv, Aq. font. ℥fs. et cras mane Sal. cathart. amar. ℥ifs. part. vicib.

9. She fweated profufely with the draught, and is much eafier this morning. Her head-ach is confiderably relieved; her pulfe foft and regular. Both dofes of the Sal. cath. amarus produced vomiting.

Capiat ftatim Ol. Ricini ℥i.—Repetatur Hauftus h. s.

10. She could not yefterday retain the oil on her ftomach, nor has yet had a ftool. She paffed an eafy night, and feels no complaint this morning, excepting great languor and laffitude, with a fenfe of weight and fulnefs in the abdomen.

Capiat ftatim Calom. gr. v. Extr. Cathart. ℈j.

11. The pills operated gently three times; her bowels are much eafier; fhe feels a flight pain of the head and general uneafinefs, as if the fit was approaching.

Incipiat cras fumere Solut. min. guttas x. ter die.

13. The fit returned on the 11th at the ufual time with great violence. The pain of her head and ftomach was alfo very fevere; fhe yet feels feels ſome pain of her ſtomach, with great reſtleſſneſs and uneaſineſs. The ſolution has not been taken till this day.

17. The paroxyſm returned at the uſual time on the 14th, when ſhe was affected with very ſevere head-ach and pains of the ſtomach and back, which ſtill continue, being accompanied with great languor. She has taken only five doſes of the ſolution ſince the 13th, and thoſe not at regular times. She was very coſtive on the 15th, when ſhe took

Calom. gr. v. c. Extr. Cathart. gr. xv.

which operated twice. She expects the paroxyſm to-day.

Repetatur Solutio.

18. The paroxyſm did not return yeſterday, until ſix P. M.; the cold ſtage was very ſevere, and attended with great pain of the ſtomach and head; but theſe ſymptoms were much relieved by two grains of opium. She ſweated profuſely during the night, and feels a ſlight head-ach and pain of the ſtomach this morning, with languor and debility. Her body is open; her pulſe natural.

Repetatur Solutio. Sumantur Opii gr. ij. urg. dolore Ventriculi.

20. She continues ſtill weak and languid; the pain of her ftomach was wholly removed by the opiate.

Repetatur Solutio.

23. She has had no return of the paroxyfm fince the 17th, and makes no complaint but of debility; fhe is, however, able to walk about, and her appetite is fomewhat better.

Omittatur Solut. min. Capiat Infus. Corticis Anguft. ℥iij ter die.

Early in October fhe had entirely recovered her health and ftrength.

